# Tetra­aqua­bis(2-methyl­benzimidazolium-1,3-diacetato-κ*O*)zinc(II) tetra­hydrate

**DOI:** 10.1107/S1600536809031766

**Published:** 2009-08-19

**Authors:** Heng-Chi Lian, Qing-Ling Ni, Xuan-Feng Jiang, Zhong-Min Cen, Jia-Huang Lin

**Affiliations:** a School of Chemistry and Chemical Engineering, Guangxi Normal University, Guilin 541004, People’s Republic of China

## Abstract

The asymmetric unit of the title compound, [Zn(C_12_H_11_N_2_O_4_)_2_(H_2_O)_4_]·4H_2_O, contains one-half of the complex mol­ecule and two uncoordin­ated water mol­ecules. The four water O atoms in the equatorial plane around the Zn^II^ centre (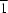 symmetry) form a distorted square-planar arrangement, while the distorted octa­hedral coordination geometry is completed by the O atoms of the zwitterionic 2-methyl­benzimidazolium-1,3-diacetate ligands in the axial positions. The benzimidazole ring system is planar, with a maximum deviation of 0.041 (3) Å. Intra­molecular O—H⋯O hydrogen bonding results in the formation of a non-planar six-membered ring. In the crystal structure, strong intra- and inter­molecular O—H⋯O hydrogen bonds link the mol­ecules into a three-dimensional network. π–π contacts between benzimidazole rings [centroid–centroid distance = 3.899 (1) Å] may further stabilize the structure.

## Related literature

For general background to metal-organic frameworks, see: Robson *et al.* (2000 ▶); Kitagawa *et al.* (2004 ▶). For a related structure, see: Ni *et al.* (2007 ▶).
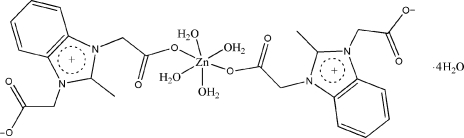


## Experimental

### 

#### Crystal data

[Zn(C_12_H_11_N_2_O_4_)_2_(H_2_O)_4_]·4H_2_O
*M* *_r_* = 703.95
Monoclinic, 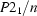

*a* = 7.2749 (9) Å
*b* = 21.265 (3) Å
*c* = 9.7794 (12) Å
β = 104.467 (2)°
*V* = 1464.9 (3) Å^3^
*Z* = 2
Mo *K*α radiation
μ = 0.92 mm^−1^
*T* = 294 K
0.32 × 0.21 × 0.15 mm

#### Data collection

Bruker SMART CCD area-detector diffractometer
Absorption correction: multi-scan (*SADABS*; Sheldrick, 1996 ▶) *T*_min_ = 0.757, *T*_max_ = 0.874
7436 measured reflections
3202 independent reflections
2582 reflections with *I* > 2σ(*I*)
*R*_int_ = 0.022

#### Refinement

*R*[*F* ^2^ > 2σ(*F* ^2^)] = 0.036
*wR*(*F* ^2^) = 0.097
*S* = 1.10
3202 reflections
237 parameters
H atoms treated by a mixture of independent and constrained refinement
Δρ_max_ = 0.98 e Å^−3^
Δρ_min_ = −0.51 e Å^−3^

### 

Data collection: *SMART* (Bruker, 1998 ▶); cell refinement: *SAINT* (Bruker, 1998 ▶); data reduction: *SAINT*; program(s) used to solve structure: *SHELXS97* (Sheldrick, 2008 ▶); program(s) used to refine structure: *SHELXL97* (Sheldrick, 2008 ▶); molecular graphics: *SHELXTL* (Sheldrick, 2008 ▶); software used to prepare material for publication: *SHELXTL*.

## Supplementary Material

Crystal structure: contains datablocks I, global. DOI: 10.1107/S1600536809031766/hk2732sup1.cif

Structure factors: contains datablocks I. DOI: 10.1107/S1600536809031766/hk2732Isup2.hkl

Additional supplementary materials:  crystallographic information; 3D view; checkCIF report

## Figures and Tables

**Table 1:** Selected geometric parameters (Å, °)

Zn1—O5	2.1023 (17)
Zn1—O6	2.1128 (16)
Zn1—O4	2.1303 (14)

**Table 2:** Hydrogen-bond geometry (Å, °)

*D*—H⋯*A*	*D*—H	H⋯*A*	*D*⋯*A*	*D*—H⋯*A*
O5—H5*B*⋯O8	0.80 (4)	1.92 (4)	2.716 (3)	170 (3)
O6—H6*A*⋯O3	0.92 (4)	1.70 (4)	2.610 (2)	170 (3)
O6—H6*B*⋯O2^ii^	0.75 (3)	2.08 (3)	2.811 (2)	164 (3)
O7—H7*A*⋯O1^iii^	0.78 (4)	2.11 (4)	2.864 (3)	165 (4)
O7—H7*B*⋯O2^iv^	0.78 (4)	2.03 (4)	2.792 (2)	167 (3)
O8—H8*A*⋯O4^iv^	0.75 (3)	2.10 (3)	2.846 (2)	177 (3)
O8—H8*B*⋯O7^v^	0.79 (3)	2.00 (3)	2.786 (3)	168 (3)

Symmetry codes: (ii) 
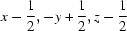
; (iii) 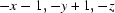; (iv) 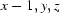; (v) 
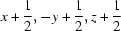
.

**Fig. 1.**
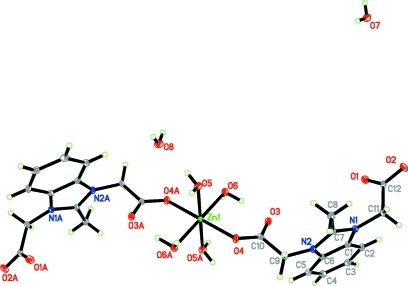
The molecular structure of the title molecule with the atom-numbering scheme. Displacement ellipsoids are drawn at the 30% probability level [symmetry code: (A) -x, -y, -z].

**Fig. 2.**
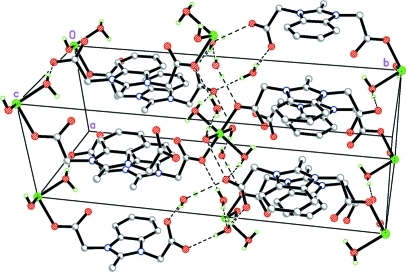
A partial packing diagram. Hydrogen bonds are shown as dashed lines.

## supplementary crystallographic information

### Comment

The quest to rational design and construct metal-organic frameworks (MOF) is highly topical, for their intriguing topologies and potential applications as functional materials in many areas such as catalysis, molecular adsorption, magnetism properties, non-linear optics and molecular sensing (Robson, 2000; Kitagawa *et al.*, 2004). In order to explore further the influence of novel polycarboxylate ligand which is a good candidate as building block on MOFs, we developed a flexible ligand 1-acetoxy-2-methylbenzimidazole-3-acetate acid [HL] (Ni *et al.*, 2007), to prepare the title mononuclear complex. We report herein its crystal structure.

The asymmetric unit of the title compound, (Fig. 1), contains one-half molecule, two coordinated and two uncoordinated water molecules. The Zn atom is surrounded by two 2-methylbenzimidazolium-1,3-diacetate and four water molecules. The four O atoms (O5, O6, O5A and O6A atoms) in the equatorial plane around the Zn atom form a distorted square-planar arrangement, while the distorted octahedral coordination is completed by the O atoms of the 2-methylbenzimidazolium-1,3-diacetate ligands (O4 and O4A) in the axial positions [symmetry code: (A) -x, -y, -z] (Table 1). The benzimidazole ring system is planar with a maximum deviation of 0.041 (3) Å for atom C7. Intramolecular O-H···O hydrogen bond results in the formation of a six-membered ring (Zn1/O3/O4/C6/C10/H6A) having twisted conformation.

In the crystal structure, strong intra- and intermolecular O-H···O hydrogen bonds (Table 2) link the molecules into a three-dimensional network, in which they may be effective in the stabilization of the structure. The π–π contact between the benzimidazole rings, Cg1—Cg2^i^ [symmetry code: (i) 1/2 + x, 1/2 - y, 1/2 + z, where Cg1 and Cg2 are centroids of the rings A (N1/N2/C1/C6/C7) and B (C1-C6), respectively] may further stabilize the structure, with centroid-centroid distance of 3.899 (1) Å.

### Experimental

After the pH of a mixture containing ZnCl_2_.2H_2_O (0.0408 g, 0.3 mmol) and ligand HL (0.0498 g, 0.2 mmol) was adjusted by ammonia to 7, the resulting solution was sealed in a Teflon-lined steel liner (25 ml) and then heated at 423 K for 3 d. Colorless block crystals were collected (yield; 28%).

### Refinement

H atoms of water molecules were located in difference Fourier maps and refined isotropically. The remaining H atoms were positioned geometrically with C-H = 0.93, 0.97 and 0.96 Å, for aromatic, methylene and methyl H atoms, respectively, and constrained to ride on their parent atoms, with U_iso_(H) = xU_eq_(C), where x = 1.5 for methyl H and x = 1.2 for all other H atoms.

### Crystal data

**Table:**

[Zn(C_12_H_11_N_2_O_4_)_2_(H_2_O)_4_]·4H_2_O	*F*(000) = 736
*M**_r_* = 703.95	*D*_x_ = 1.596 Mg m^−^^3^
Monoclinic, *P*2_1_/*n*	Mo *K*α radiation, λ = 0.71073 Å
Hall symbol: -P 2yn	Cell parameters from 3729 reflections
*a* = 7.2749 (9) Å	θ = 2.4–27.0°
*b* = 21.265 (3) Å	µ = 0.92 mm^−^^1^
*c* = 9.7794 (12) Å	*T* = 294 K
β = 104.467 (2)°	Block, colorless
*V* = 1464.9 (3) Å^3^	0.32 × 0.21 × 0.15 mm
*Z* = 2	

### Data collection

**Table:**

Bruker SMART CCD area-detector diffractometer	3202 independent reflections
Radiation source: fine-focus sealed tube	2582 reflections with *I* > 2σ(*I*)
graphite	*R*_int_ = 0.022
ω scans	θ_max_ = 27.1°, θ_min_ = 1.9°
Absorption correction: multi-scan (*SADABS*; Sheldrick, 1996)	*h* = −8→9
*T*_min_ = 0.757, *T*_max_ = 0.874	*k* = −14→27
7436 measured reflections	*l* = −10→12

### Refinement

**Table:**

Refinement on *F*^2^	Primary atom site location: structure-invariant direct methods
Least-squares matrix: full	Secondary atom site location: difference Fourier map
*R*[*F*^2^ > 2σ(*F*^2^)] = 0.036	Hydrogen site location: inferred from neighbouring sites
*wR*(*F*^2^) = 0.097	H atoms treated by a mixture of independent and constrained refinement
*S* = 1.10	*w* = 1/[σ^2^(*F*_o_^2^) + (0.0541*P*)^2^ + 0.4211*P*] where *P* = (*F*_o_^2^ + 2*F*_c_^2^)/3
3202 reflections	(Δ/σ)_max_ < 0.001
237 parameters	Δρ_max_ = 0.98 e Å^−^^3^
0 restraints	Δρ_min_ = −0.51 e Å^−^^3^

### Special details

**Table:**

Geometry. All e.s.d.'s (except the e.s.d. in the dihedral angle between two l.s. planes) are estimated using the full covariance matrix. The cell e.s.d.'s are taken into account individually in the estimation of e.s.d.'s in distances, angles and torsion angles; correlations between e.s.d.'s in cell parameters are only used when they are defined by crystal symmetry. An approximate (isotropic) treatment of cell e.s.d.'s is used for estimating e.s.d.'s involving l.s. planes.
Refinement. Refinement of *F*^2^ against ALL reflections. The weighted *R*-factor *wR* and goodness of fit *S* are based on *F*^2^, conventional *R*-factors *R* are based on *F*, with *F* set to zero for negative *F*^2^. The threshold expression of *F*^2^ > σ(*F*^2^) is used only for calculating *R*-factors(gt) *etc*. and is not relevant to the choice of reflections for refinement. *R*-factors based on *F*^2^ are statistically about twice as large as those based on *F*, and *R*- factors based on ALL data will be even larger.

### Fractional atomic coordinates and isotropic or equivalent isotropic displacement parameters (Å^2^)

**Table:**

	*x*	*y*	*z*	*U*_iso_*/*U*_eq_	
Zn1	0.0000	0.0000	0.0000	0.01544 (12)	
O1	0.1738 (2)	0.37790 (7)	−0.04309 (16)	0.0235 (4)	
O2	0.3049 (2)	0.44774 (7)	0.12371 (16)	0.0225 (4)	
O3	0.1638 (2)	0.13757 (7)	−0.05157 (16)	0.0203 (3)	
O4	0.2586 (2)	0.05044 (7)	0.07499 (16)	0.0190 (3)	
O5	−0.0725 (3)	0.01888 (8)	0.19098 (18)	0.0210 (4)	
H5A	−0.012 (5)	−0.0003 (15)	0.242 (4)	0.042 (11)*	
H5B	−0.180 (5)	0.0100 (13)	0.190 (3)	0.030 (8)*	
O6	−0.1608 (2)	0.07925 (7)	−0.08958 (19)	0.0201 (3)	
H6A	−0.054 (5)	0.1041 (17)	−0.080 (4)	0.065 (11)*	
H6B	−0.191 (4)	0.0718 (15)	−0.167 (3)	0.038 (9)*	
O7	−0.8479 (3)	0.55949 (9)	−0.00644 (19)	0.0270 (4)	
H7A	−0.940 (6)	0.5704 (19)	0.012 (3)	0.060 (12)*	
H7B	−0.814 (5)	0.5299 (17)	0.040 (3)	0.041 (9)*	
O8	−0.4182 (3)	−0.02154 (9)	0.2126 (2)	0.0255 (4)	
H8A	−0.505 (5)	−0.0026 (12)	0.179 (3)	0.021 (7)*	
H8B	−0.409 (4)	−0.0284 (15)	0.294 (3)	0.036 (9)*	
N1	0.4887 (2)	0.29835 (8)	0.03733 (19)	0.0149 (4)	
N2	0.4950 (2)	0.19548 (8)	0.03276 (18)	0.0145 (4)	
C1	0.4865 (3)	0.28122 (10)	−0.1007 (2)	0.0157 (4)	
C2	0.4759 (3)	0.31727 (10)	−0.2218 (2)	0.0197 (5)	
H2A	0.4753	0.3610	−0.2199	0.024*	
C3	0.4664 (3)	0.28382 (11)	−0.3447 (2)	0.0230 (5)	
H3A	0.4599	0.3059	−0.4279	0.028*	
C4	0.4661 (3)	0.21788 (11)	−0.3484 (2)	0.0225 (5)	
H4A	0.4567	0.1976	−0.4341	0.027*	
C5	0.4795 (3)	0.18208 (10)	−0.2272 (2)	0.0194 (5)	
H5C	0.4811	0.1384	−0.2286	0.023*	
C6	0.4903 (3)	0.21592 (10)	−0.1039 (2)	0.0152 (4)	
C7	0.4906 (3)	0.24587 (9)	0.1143 (2)	0.0147 (4)	
C8	0.4841 (3)	0.24357 (10)	0.2639 (2)	0.0198 (5)	
H8C	0.4816	0.2856	0.2992	0.030*	
H8D	0.5944	0.2221	0.3181	0.030*	
H8E	0.3720	0.2215	0.2718	0.030*	
C9	0.4878 (3)	0.12953 (9)	0.0736 (2)	0.0158 (4)	
H9A	0.5342	0.1257	0.1752	0.019*	
H9B	0.5697	0.1048	0.0301	0.019*	
C10	0.2853 (3)	0.10399 (9)	0.0281 (2)	0.0148 (4)	
C11	0.4965 (3)	0.36335 (9)	0.0885 (2)	0.0177 (4)	
H11A	0.5867	0.3866	0.0498	0.021*	
H11B	0.5441	0.3631	0.1905	0.021*	
C12	0.3065 (3)	0.39821 (9)	0.0510 (2)	0.0161 (4)	

### Atomic displacement parameters (Å^2^)

**Table:**

	*U*^11^	*U*^22^	*U*^33^	*U*^12^	*U*^13^	*U*^23^
Zn1	0.01524 (18)	0.01194 (17)	0.01867 (19)	−0.00014 (13)	0.00336 (13)	0.00262 (13)
O1	0.0199 (8)	0.0220 (8)	0.0262 (9)	0.0010 (6)	0.0010 (7)	−0.0064 (7)
O2	0.0242 (8)	0.0161 (7)	0.0239 (8)	0.0047 (6)	−0.0003 (6)	−0.0044 (6)
O3	0.0157 (7)	0.0157 (7)	0.0272 (8)	−0.0005 (6)	0.0012 (6)	0.0060 (6)
O4	0.0188 (8)	0.0136 (7)	0.0232 (8)	−0.0020 (6)	0.0026 (6)	0.0051 (6)
O5	0.0179 (9)	0.0242 (8)	0.0203 (8)	0.0002 (7)	0.0038 (7)	0.0043 (7)
O6	0.0195 (8)	0.0168 (8)	0.0228 (9)	−0.0008 (6)	0.0029 (7)	0.0020 (7)
O7	0.0241 (10)	0.0240 (9)	0.0343 (10)	0.0040 (7)	0.0100 (8)	0.0077 (8)
O8	0.0202 (9)	0.0270 (9)	0.0290 (10)	0.0038 (8)	0.0056 (8)	0.0048 (8)
N1	0.0157 (8)	0.0103 (8)	0.0185 (9)	−0.0002 (7)	0.0041 (7)	−0.0001 (7)
N2	0.0150 (8)	0.0112 (8)	0.0166 (9)	−0.0021 (7)	0.0025 (7)	0.0012 (7)
C1	0.0128 (10)	0.0168 (10)	0.0173 (10)	0.0001 (8)	0.0033 (8)	0.0002 (8)
C2	0.0187 (10)	0.0161 (10)	0.0243 (12)	−0.0007 (8)	0.0053 (9)	0.0055 (9)
C3	0.0211 (11)	0.0289 (12)	0.0203 (11)	0.0016 (9)	0.0080 (9)	0.0073 (9)
C4	0.0219 (11)	0.0276 (12)	0.0192 (11)	0.0013 (9)	0.0073 (9)	−0.0015 (9)
C5	0.0191 (11)	0.0176 (11)	0.0223 (11)	0.0001 (8)	0.0065 (9)	−0.0015 (9)
C6	0.0131 (10)	0.0157 (10)	0.0170 (10)	−0.0001 (8)	0.0040 (8)	0.0014 (8)
C7	0.0110 (9)	0.0132 (10)	0.0184 (10)	−0.0004 (8)	0.0011 (8)	−0.0005 (8)
C8	0.0212 (11)	0.0194 (11)	0.0178 (11)	−0.0007 (9)	0.0033 (9)	−0.0012 (8)
C9	0.0172 (10)	0.0095 (9)	0.0202 (11)	0.0005 (8)	0.0036 (8)	0.0018 (8)
C10	0.0173 (10)	0.0141 (10)	0.0136 (10)	0.0007 (8)	0.0048 (8)	−0.0023 (8)
C11	0.0190 (11)	0.0119 (9)	0.0217 (11)	0.0002 (8)	0.0038 (8)	−0.0032 (8)
C12	0.0175 (10)	0.0131 (10)	0.0181 (10)	0.0003 (8)	0.0052 (8)	0.0017 (8)

### Geometric parameters (Å, °)

**Table:**

Zn1—O5	2.1023 (17)	N2—C6	1.398 (3)
Zn1—O5^i^	2.1023 (17)	N2—C9	1.463 (2)
Zn1—O6^i^	2.1128 (16)	C1—C6	1.390 (3)
Zn1—O6	2.1128 (16)	C1—C2	1.396 (3)
Zn1—O4	2.1303 (14)	C2—C3	1.384 (3)
Zn1—O4^i^	2.1303 (14)	C2—H2A	0.9300
O1—C12	1.233 (3)	C3—C4	1.402 (3)
O2—C12	1.273 (2)	C3—H3A	0.9300
O3—C10	1.247 (2)	C4—C5	1.392 (3)
O4—C10	1.261 (2)	C4—H4A	0.9300
O5—H5A	0.71 (3)	C5—C6	1.389 (3)
O5—H5B	0.80 (4)	C5—H5C	0.9300
O6—H6A	0.92 (4)	C7—C8	1.476 (3)
O6—H6B	0.75 (3)	C8—H8C	0.9600
O7—H7A	0.78 (4)	C8—H8D	0.9600
O7—H7B	0.78 (4)	C8—H8E	0.9600
O8—H8A	0.75 (3)	C9—C10	1.528 (3)
O8—H8B	0.79 (3)	C9—H9A	0.9700
N1—C7	1.345 (3)	C9—H9B	0.9700
N1—C1	1.394 (3)	C11—C12	1.531 (3)
N1—C11	1.466 (3)	C11—H11A	0.9700
N2—C7	1.341 (3)	C11—H11B	0.9700
			
O5—Zn1—O5^i^	180.00 (14)	C4—C3—H3A	118.8
O5—Zn1—O6^i^	91.13 (7)	C5—C4—C3	121.7 (2)
O5^i^—Zn1—O6^i^	88.87 (7)	C5—C4—H4A	119.2
O5—Zn1—O6	88.87 (7)	C3—C4—H4A	119.2
O5^i^—Zn1—O6	91.13 (7)	C6—C5—C4	115.6 (2)
O6^i^—Zn1—O6	180.00 (9)	C6—C5—H5C	122.2
O5—Zn1—O4	89.65 (7)	C4—C5—H5C	122.2
O5^i^—Zn1—O4	90.35 (7)	C5—C6—C1	122.7 (2)
O6^i^—Zn1—O4	84.78 (6)	C5—C6—N2	130.64 (19)
O6—Zn1—O4	95.22 (6)	C1—C6—N2	106.58 (18)
O5—Zn1—O4^i^	90.35 (7)	N2—C7—N1	109.18 (18)
O5^i^—Zn1—O4^i^	89.65 (7)	N2—C7—C8	125.07 (19)
O6^i^—Zn1—O4^i^	95.22 (6)	N1—C7—C8	125.74 (19)
O6—Zn1—O4^i^	84.78 (6)	C7—C8—H8C	109.5
O4—Zn1—O4^i^	180.00 (12)	C7—C8—H8D	109.5
C10—O4—Zn1	122.18 (13)	H8C—C8—H8D	109.5
Zn1—O5—H5A	106 (3)	C7—C8—H8E	109.5
Zn1—O5—H5B	114 (2)	H8C—C8—H8E	109.5
H5A—O5—H5B	108 (4)	H8D—C8—H8E	109.5
Zn1—O6—H6A	93 (2)	N2—C9—C10	111.03 (16)
Zn1—O6—H6B	104 (2)	N2—C9—H9A	109.4
H6A—O6—H6B	104 (3)	C10—C9—H9A	109.4
H7A—O7—H7B	106 (4)	N2—C9—H9B	109.4
H8A—O8—H8B	113 (3)	C10—C9—H9B	109.4
C7—N1—C1	108.75 (17)	H9A—C9—H9B	108.0
C7—N1—C11	126.66 (18)	O3—C10—O4	126.52 (19)
C1—N1—C11	124.53 (17)	O3—C10—C9	117.38 (18)
C7—N2—C6	108.77 (17)	O4—C10—C9	116.09 (17)
C7—N2—C9	126.57 (17)	N1—C11—C12	114.77 (17)
C6—N2—C9	124.43 (17)	N1—C11—H11A	108.6
C6—C1—N1	106.69 (18)	C12—C11—H11A	108.6
C6—C1—C2	121.8 (2)	N1—C11—H11B	108.6
N1—C1—C2	131.5 (2)	C12—C11—H11B	108.6
C3—C2—C1	115.8 (2)	H11A—C11—H11B	107.6
C3—C2—H2A	122.1	O1—C12—O2	126.3 (2)
C1—C2—H2A	122.1	O1—C12—C11	120.08 (18)
C2—C3—C4	122.4 (2)	O2—C12—C11	113.60 (18)
C2—C3—H3A	118.8		
			
O5—Zn1—O4—C10	−107.15 (16)	C7—N2—C6—C1	1.1 (2)
O5^i^—Zn1—O4—C10	72.85 (16)	C9—N2—C6—C1	175.92 (17)
O6^i^—Zn1—O4—C10	161.69 (16)	C6—N2—C7—N1	−1.8 (2)
O6—Zn1—O4—C10	−18.31 (16)	C9—N2—C7—N1	−176.41 (17)
C7—N1—C1—C6	−1.0 (2)	C6—N2—C7—C8	176.87 (19)
C11—N1—C1—C6	176.50 (17)	C9—N2—C7—C8	2.2 (3)
C7—N1—C1—C2	176.8 (2)	C1—N1—C7—N2	1.7 (2)
C11—N1—C1—C2	−5.7 (3)	C11—N1—C7—N2	−175.70 (17)
C6—C1—C2—C3	1.0 (3)	C1—N1—C7—C8	−176.92 (19)
N1—C1—C2—C3	−176.5 (2)	C11—N1—C7—C8	5.7 (3)
C1—C2—C3—C4	0.4 (3)	C7—N2—C9—C10	96.0 (2)
C2—C3—C4—C5	−1.4 (3)	C6—N2—C9—C10	−77.8 (2)
C3—C4—C5—C6	0.9 (3)	Zn1—O4—C10—O3	9.4 (3)
C4—C5—C6—C1	0.5 (3)	Zn1—O4—C10—C9	−170.20 (13)
C4—C5—C6—N2	176.3 (2)	N2—C9—C10—O3	9.8 (3)
N1—C1—C6—C5	176.56 (18)	N2—C9—C10—O4	−170.54 (17)
C2—C1—C6—C5	−1.5 (3)	C7—N1—C11—C12	−103.4 (2)
N1—C1—C6—N2	−0.1 (2)	C1—N1—C11—C12	79.6 (2)
C2—C1—C6—N2	−178.15 (18)	N1—C11—C12—O1	−17.0 (3)
C7—N2—C6—C5	−175.2 (2)	N1—C11—C12—O2	163.61 (18)
C9—N2—C6—C5	−0.4 (3)		

Symmetry codes: (i) −*x*, −*y*, −*z*.

### Hydrogen-bond geometry (Å, °)

**Table:**

*D*—H···*A*	*D*—H	H···*A*	*D*···*A*	*D*—H···*A*
O5—H5B···O8	0.80 (4)	1.92 (4)	2.716 (3)	170 (3)
O6—H6A···O3	0.92 (4)	1.70 (4)	2.610 (2)	170 (3)
O6—H6B···O2^ii^	0.75 (3)	2.08 (3)	2.811 (2)	164 (3)
O7—H7A···O1^iii^	0.78 (4)	2.11 (4)	2.864 (3)	165 (4)
O7—H7B···O2^iv^	0.78 (4)	2.03 (4)	2.792 (2)	167 (3)
O8—H8A···O4^iv^	0.75 (3)	2.10 (3)	2.846 (2)	177 (3)
O8—H8B···O7^v^	0.79 (3)	2.00 (3)	2.786 (3)	168 (3)

Symmetry codes: (ii) *x*−1/2, −*y*+1/2, *z*−1/2; (iii) −*x*−1, −*y*+1, −*z*; (iv) *x*−1, *y*, *z*; (v) *x*+1/2, −*y*+1/2, *z*+1/2.
